# Acidosis is associated with lower insulin sensitivity and incident type 2 diabetes in indigenous Americans: A prospective cohort study

**DOI:** 10.1111/dom.70037

**Published:** 2025-08-18

**Authors:** Andrés M. Treviño‐Alvarez, Tomás Cabeza de Baca, Emma J. Stinson, Marci E. Gluck, Paolo Piaggi, Jonathan Krakoff, Douglas C. Chang

**Affiliations:** ^1^ Obesity and Diabetes Clinical Research Section, Phoenix Epidemiology and Clinical Research Branch, National Institute of Diabetes and Digestive and Kidney Diseases National Institutes of Health Phoenix Arizona USA; ^2^ Department of Neurology Universidad Autonoma de Nuevo Leon Monterrey México; ^3^ Department of Information Engineering University of Pisa Pisa Italy

**Keywords:** anion gap, bicarbonate, insulin resistance, type 2 diabetes

## Abstract

**Aim:**

Whether markers of acid accumulation [decreased plasma bicarbonate, increased anion gap (AG) and corrected anion gap (CAG)] are associated with insulin sensitivity and alter diabetes risk is unclear. We aimed to examine the association of these markers with the gold‐standard measure of insulin sensitivity and incident type 2 diabetes.

**Materials and Methods:**

Healthy adults without diabetes enrolled in a longitudinal study had baseline measures of acidosis (bicarbonate, AG, CAG), insulin sensitivity by hyperinsulinaemic‐euglycaemic clamp, and body composition. In cross‐sectional analysis (*n* = 296, 188 male), the relationship between acid markers and insulin sensitivity (M‐low) was evaluated. Progression to type 2 diabetes was evaluated by Cox regression models (per 1‐SD change) in those with appropriate follow‐up visits (*n* = 233).

**Results:**

Increased AG and CAG were associated with lower M‐low, adjusting for age, sex, body fat (%) and estimated glomerular filtration rate (AG: partial *r* = −0.22, CAG: partial *r* = −0.24, both *p* < 0.0001) but not with bicarbonate. During a median follow‐up time of 8.0 years, 50 participants developed type 2 diabetes. CAG was associated with increased risk of type 2 diabetes (HR: 1.32, 95% CI 1.01–1.74, *p* = 0.044) in a model adjusted for age, sex, body fat (%) and baseline plasma glucose, but not after further adjusting for M‐low (*p* = 0.17). Bicarbonate and AG were not associated with risk of diabetes.

**Conclusion:**

Acidosis was associated with lower insulin sensitivity and increased risk of type 2 diabetes that was attenuated by measures of lower insulin sensitivity. Acidosis as reflected by CAG may be a target for intervention.

## 1 INTRODUCTION

Insulin resistance is linked with important health conditions including type 2 diabetes, obesity, gallstones, chronic kidney disease, metabolic dysfunction associated with fatty liver disease and cardiovascular disease.^1^, ^2^, ^3^ Insulin resistance (alongside relatively diminished insulin secretion) is a major pathophysiological risk factor for type 2 diabetes^4^ underlying the importance of understanding contributing factors.

Prior evidence suggests that acidosis may lead to insulin resistance. Most hormones, including insulin, interact with their cell receptors primarily in the interstitial space. Unlike blood, which contains substantial concentrations of haemoglobin and albumin to buffer pH, the interstitial space has much less capacity to buffer acidotic states.^5^ A rat model of type 2 diabetes demonstrated reduced interstitial pH.^6^ Acidosis has been shown to inhibit the rate‐limiting enzyme of glycolysis (phosphofructokinase), decrease insulin binding to its receptor in adipocytes, and reduce phosphorylation of Akt and insulin sensitivity in rat muscle.^7^, ^8^, ^9^, ^10^ In patients with chronic kidney disease, which is associated with both metabolic acidosis and insulin resistance, correction of metabolic acidosis improves insulin resistance.^11^ Defronzo and Beckles experimentally induced a state of chronic metabolic acidosis by administering ammonium chloride to a small number of healthy research participants and demonstrated a reduction of insulin sensitivity using the glucose‐clamp technique, a gold standard for measuring insulin sensitivity.^12^ Importantly, the change in insulin sensitivity was observed with only a mild degree of acidosis (serum bicarbonate decreased from 24 to 19 mEq/L).

In the U.S. National Health and Nutrition Examination Survey, lower bicarbonate and higher anion gap, indicating acid accumulation, were associated with fasting insulin, a surrogate measure of insulin resistance.^13^ In prospective studies, lower serum bicarbonate and higher anion gap were associated with the risk of progression to diabetes.^14^, ^15^, ^16^ However, there remain important gaps in the scientific literature. It is unclear whether bicarbonate and anion gap are associated with insulin resistance as measured by the hyperinsulinaemic‐euglycaemic clamp technique, as prior studies relied upon surrogate measures of insulin sensitivity instead of reference methods. Furthermore, it is unknown whether these markers of acid accumulation are associated with incident diabetes independent of insulin resistance.

In the current study, we first investigated in healthy adults without diabetes whether markers of acid accumulation (plasma bicarbonate, anion gap (AG) and corrected anion gap (CAG)) were cross‐sectionally associated with whole‐body and hepatic insulin sensitivity, measured with a 2‐step hyperinsulinaemic‐euglycaemic clamp in combination with a glucose tracer. We then investigated prospectively whether these acid accumulation markers were associated with the development of type 2 diabetes, adjusting for important covariates including insulin sensitivity.

## 2 METHODS

Indigenous American adults living in the southwestern US, who did not have diabetes, were invited to participate in an inpatient study involving detailed metabolic phenotyping to investigate risk factors for diabetes (NCT00340132).^4^, ^17^ After discharge from this inpatient study, participants were followed for the development of diabetes in an outpatient longitudinal cohort study (NCT00339482).^18^

Both study protocols were approved by the Institutional Review Board of the National Institutes of Health and the participating Tribe. All volunteers provided informed consent.

### 2.1 Detailed metabolic phenotyping at baseline

During the inpatient stay (baseline) at the Clinical Research Unit (Phoenix, Arizona), participants were screened by medical history, physical examination and routine laboratory tests to ensure they were healthy. None of the females were pregnant and no participants were on medications. Screening labs included acid accumulation markers (plasma bicarbonate, AG and CAG – see below for calculations) and serum creatinine. In this secondary analysis, only those with estimated glomerular filtration rate (eGFR) greater than or equal to 60 mL/min per 1.73 m^2^ based on the 2009 CKD‐EPI equation were included.^19^ Participants who passed screening were admitted and placed on a weight‐maintaining diet (50% carbohydrate, 30% fat and 20% protein). After at least 3 days of this diet, participants had a 75 g oral glucose tolerance test (OGTT) to verify the absence of diabetes at baseline (fasting plasma glucose <126 mg/dL and 2‐h plasma glucose <200 mg/dL). Phenotyping procedures further included body composition measurements and a 2‐step hyperinsulinaemic‐euglycaemic clamp for insulin sensitivity (see detailed description below).

### 2.2 Longitudinal follow‐up for diabetes

After discharge from the inpatient stay, volunteers were invited to follow up for the development of diabetes through outpatient examinations every 2 years. These outpatient examinations included a review of medical records and a 75 g OGTT to diagnose diabetes. The assigned date of diabetes diagnosis was based on the OGTT date if diagnosed during this outpatient visit. If diagnosed outside the outpatient visit (e.g., diagnosed at a hospital or by an outside physician), the assigned date of diabetes diagnosis was based on the review of medical records.

### 2.3 Acid accumulation markers

Bicarbonate, sodium, chloride, creatinine and albumin were obtained after an overnight fast and measured in the hospital laboratory (Dade Behring Dimension RxL Chemistry analyser, Siemens Medical Solutions, Malvern, PA). Traditional AG was calculated as follows: AG = [Na]−([Cl] + [HCO3]). The equation for CAG was as follows: CAG = traditional anion gap + (4.4 ‐ serum albumin[g/dL]) X 2.5.^20^

### 2.4 Body composition

Body composition was measured by dual‐energy X‐ray absorptiometry (DXA; DPX‐L and Lunar Prodigy, GE Lunar, Madison, WI) or by underwater weighing in which residual lung volume was determined using helium dilution.^21^ To make these measurements comparable, previously derived equations were applied.^21^, ^22^

### 2.5 Two‐step hyperinsulinaemic‐euglycaemic clamp

Insulin resistance at physiologic and supra‐physiologic insulin concentrations was assessed by two‐step hyperinsulinaemic‐euglycaemic clamp as previously described in detail.^17^, ^23^ Briefly, the hyperinsulinaemic‐euglycaemic clamp was performed after an overnight fast, in which a primed continuous insulin infusion (240 pmol/m^2^ body surface area/min) was administered for 100 min, followed by a second 100 min at 2400 pmol/m^2^ body surface area/min. During both steps, a 20% dextrose solution was infused at varying rates to maintain a plasma glucose level of 100 mg/dL. The glucose uptake rates (M‐low and M‐high) were calculated for the last 40 min of each step of the insulin infusion, corrected for the steady‐state plasma glucose level, the steady‐state insulin concentration (M‐low only), and standardised to account for metabolic body size, which is calculated as fat‐free mass + 17.7 kg.^24^ Hepatic glucose production (HGP) was determined at baseline prior to insulin infusion (HGP‐basal), and at the end of the low‐dose insulin infusion (HGP‐insulin) using a primed (30 μCi), continuous (0.3 μCi/min) infusion of [3‐H^3^]‐glucose, calculated by the Steele equation.^25^ In the calculation of M‐low, residual HGP during the insulin infusion (i.e., HGP‐insulin) was accounted for.

Plasma glucose concentrations were determined by the glucose oxidase method (Beckman Instruments, Fullerton, CA) and plasma insulin concentrations by the Herbert modification.^26^, ^27^ Values from subsequent assays were regressed to the original radioimmunoassay using comparative equations.

### 2.6 Statistical analysis

All analyses were performed using SAS 9.4 (SAS Institute Inc., Cary, NC, USA). Baseline characteristics were compared between participants who subsequently developed diabetes and those who did not. Normally distributed data were presented using mean ± standard deviation, whereas skewed data were presented as the median and interquartile ranges. The glucose uptake values (M‐low and M‐high) were log_10_ transformed to approximate a normal distribution. Differences between progressors to diabetes and non‐progressors were assessed using independent sample *t* tests for continuous variables and chi‐square tests for categorical variables.

The prospective relationship between acid accumulation markers (bicarbonate, AG and CAG) and the risk of type 2 diabetes was assessed using Cox proportional hazards models. Participants were censored at the date of diabetes diagnosis (via OGTT assessment or medical record review), or to the last outpatient exam date during follow‐up. Cox proportional hazards models were progressively adjusted as follows: (1) unadjusted; (2) adjusted for age, sex and body fat (%); (3) further adjusted for baseline 2‐h plasma glucose; (4) further adjusted for M‐low. In the final model, M‐low was chosen as the measure of insulin resistance over M‐high as M‐low was previously shown to be more strongly associated with the risk of diabetes.^23^ All continuous variables were standardised to mean = 0 and SD = 1. Proportional hazards assumptions were assessed via Martingale residuals.

## 3 RESULTS

### 3.1 Cross‐sectional analysis: Acid accumulation is associated with lower insulin sensitivity

Characteristics of 188 male and 108 female participants in the cross‐sectional analysis are shown in Table 1. Mean age was 29.3 ± 7.0 years and mean BMI was 33.5 ± 7.2 kg/m^2^.

**TABLE 1:** Participant characteristics for cross‐sectional analysis.

	Total *n* = 296	Female *n* = 108	Male *n* = 188
Age (years), *n* = 296	29.3 ± 7.0	29.2 ± 6.8	29.4 ± 7.2
Body weight (kg), *n* = 296	94.6 ± 21.7	89.9 ± 20.5	97.3 ± 22.0
BMI (kg/m^2^) *n* = 296	33.5 ± 7.2	34.9 ± 7.6	32.6 ± 6.9
Fat mass (kg) *n* = 296	31.4 ± 12.1	35.6 ± 11.6	28.9 ± 11.7
Fat‐free mass (kg), *n* = 296	63.2 ± 12.9	54.3 ± 10.0	68.4 ± 11.5
Body fat (%), *n* = 296	32.4 ± 7.7	38.8 ± 5.9	28.7 ± 6.1
Fasting plasma glucose(mg/dL), *n* = 296	85.3 ± 10.4	88.4 ± 11.0	83.6 ± 9.7
2‐h plasma glucose (mg/dL), *n* = 296	117.2 ± 32.2	128.3 ± 30.8	110.8 ± 31.3
eGFR (mL/min per 1.73 m^2^), *n* = 296	101.2 ± 16.5	99.8 ± 17.4	102.1 ± 15.9
Bicarbonate (mEq/L), *n* = 296	25.9 ± 2.4	25.1 ± 2.4	26.3 ± 2.4
Anion gap (mEq/L), *n* = 296	7.6 ± 3.1	7.5 ± 3.0	7.7 ± 3.2
Corrected anion gap (mEq/L), *n* = 294	8.6 ± 3.3	8.9 ± 3.3	8.4 ± 3.3
M‐low (mg/kg EMBS/min, *n* = 294	0.41 ± 0.14	0.41 ± 0.13	0.41 ± 0.13
M‐high (mg/kg EMBS/min), *n* = 281	0.92 ± 0.13	0.93 ± 0.11	0.92 ± 0.13
HGP‐basal (mg/kg FFM/min), *n* = 293	2.47 ± 0.39	2.62 ± 0.43	2.39 ± 0.34
HGP‐insulin (mg/kg FFM/min), *n* = 294	0.46 ± 0.49	0.44 ± 0.49	0.47 ± 0.50

*Note*: All values expressed as mean ± SD. BMI, body mass index; eGFR, estimated glomerular filtration rate. Log_10_ values of M‐low and M‐high; HGP‐basal, hepatic glucose production during the basal period (prior to insulin infusion); HGP‐insulin, hepatic glucose production during the insulin infusion.

**FIGURE 1:**
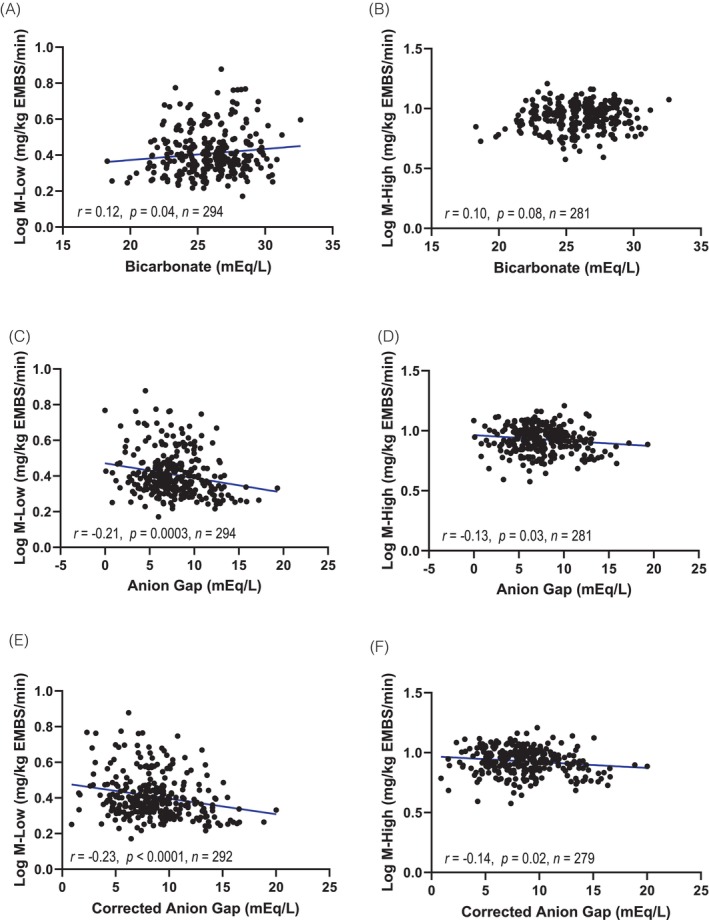
Pearson correlation coefficients of markers of metabolic acidosis with M‐low and M‐high (log_10_). All values are adjusted by age, sex, body fat (%) and estimated glomerular filtration rate. Means were added into the residuals to retain original variable units. (A) Bicarbonate was positively associated with adjusted M‐low (*r* = 0.12, *p* = 0.04); (B) there was no significant association between bicarbonate and m‐high (*r* = 0.10, *p* = 0.08). Adjusted anion gap was negatively associated with both (C) M‐low and (D) M‐high (*r* = −20, *p* = 0.0003; *r* = −0.13, *p* = 0.03, respectively). Adjusted corrected anion gap was (E) negatively associated with adjusted M‐low (*r* = −0.23, *p* < 0.0001) and m‐high (*r* = −0.14, *p* = 0.02).

Lower bicarbonate was associated with lower whole‐body insulin sensitivity (M‐low, *r* = 0.12, *p =* 0.04; M‐high, *r* = 0.12, *p* = 0.049). After adjusting for covariates (age, sex, body fat (%), and eGFR), lower bicarbonate remained associated with lower M‐low (*r* = 0.12, *p* = 0.04, Figure 1A) but not M‐high (*r* = 0.10, *p* = 0.08, Figure 1B).

AG was correlated with M‐low (*r* = −0.14, *p* = 0.01) but not M‐high (*r* = 0.10, *p* = 0.11). However, after adjusting for covariates, AG was associated with both M‐low (*r* = −0.21, *p* = 0.0003, Figure 1C) and M‐high (*r* = −0.13, *p* = 0.03, Figure 1D). Similarly, higher CAG was significantly associated with lower whole‐body insulin sensitivity, as reflected by both unadjusted and adjusted correlations for M‐low (unadjusted *r* = −0.19; *p* = 0.0009; adjusted *r* = −0.23, *p* < 0.0001, Figure 1E) and M‐high (unadjusted *r* = −0.12, *p* = 0.038; adjusted *r* = −0.14, *p* = 0.02, Figure 1F).

Bicarbonate, AG and CAG were not associated with HGP‐insulin, a measure of liver‐specific insulin sensitivity, when adjusted for these confounders (adjusted *r* = −0.05, *p* = 0.41; adjusted *r* = −0.03, *p* = 0.62; adjusted *r* = 0.001; *p* = 0.98).

### 3.2 Prospective analysis: Acid accumulation and incident type 2 diabetes

Of the 296 participants in the cross‐sectional analysis, 233 contributed a median follow‐up time of 8.0 years (IQR 4.8–10.8 years). Of these 233 participants who contributed follow‐up, 50 (21%) progressed to develop type 2 diabetes. Baseline characteristics of these 233 participants are shown in Table 2. As expected, those who developed type 2 diabetes had higher weight, BMI, fat mass, body fat percentage, and plasma glucose concentrations during the OGTT, and lower insulin sensitivity (all *p*'s < 0.05; Table 2). Acid accumulation markers of those who developed type 2 diabetes were not significantly different at baseline. However, there was a trend towards higher CAG in those who later developed type 2 diabetes (*p* = 0.06).

**TABLE 2:** Baseline characteristics of participants who did (+) and did not (−) develop type 2 diabetes.

Variable	Total	(+) Type 2 diabetes	(−) Type 2 diabetes
*n* (%)	233	50 (21%)	183 (79%)
Demographics			
Age (year)	28.8 (6.8)	30.4 (7.4)	28.4 (6.6)
Female sex, *n* (%)	94 (40%)	26 (52%)	68 (37%)
Body composition			
Weight (kg)	94.2 (22.1)	102.7 (22.6)**	91.9 (21.5)
BMI (kg/m^2^)	33.5 (7.2)	37.0 (6.8)***	32.6 (7.1)
Fat mass (kg)	31.5 (12.1)	37.1 (11.5)***	30.0 (11.8)
Fat‐free mass (kg)	62.8 (13.3)	65.6 (14.0)	62.0 (13.0)
Body fat (%)	32.6 (7.8)	35.7 (6.2)**	31.8 (8.0)
Oral glucose tolerance test			
Fasting plasma glucose (mg/dL)	85.5 (10.3)	89.4 (12.5)*	84.4 (9.4)
2‐h plasma glucose (mg/dL)	116.9 (32.3)	134.6 (35.9)***	112.1 (29.6)
Acid markers			
Bicarbonate (mEq/L)	25.8 (2.4)	25.4 (2.4)	25.9 (2.4)
Anion gap (mEq/L)	7.4 (3.2)	7.9 (3.7)	7.3 (3.0)
Corrected anion gap (mEq/L)	8.4 (3.4)	9.2 (3.8)	8.2 (3.2)
HIEC			
M‐low (mg/kg EMBS/min)^a^	0.41 (0.15)	0.34 (0.10)***	0.43 (0.15)
M‐high (mg/kg EMBS/min)^b^	0.93 (0.12)	0.89 (0.11)*	0.94 (0.12)
HGP‐basal (mg/kg FFM/min)	2.50 (0.39)	2.49 (0.42)	2.50 (0.38)
HGP‐insulin (mg/kg FFM/min)	0.48 (0.50)	0.55 (0.50)	0.46 (0.42)
Follow up			
Total follow‐up time (years)^b^	8.0 (4.8–10.8)	5.7 (3.2–8.4)***	8.6 (5.6–11.4)

*Note*: Values are expressed as means ± SD or *n* (%) unless specified otherwise.

Abbreviations: EMBS, estimated metabolic body size calculated as, fat‐free mass + 17.7 kg; HGP‐basal, hepatic glucose production during the basal period (prior to insulin infusion); HGP‐insulin, hepatic glucose production during the insulin infusion; HIEC, hyperinsulinaemic‐euglycaemic clamp; OGTT, oral glucose tolerance test.

^a^ Log_10_.

^b^ Median (IQR).

* *p* < 0.05.

** *p* < 0.01.

*** *p* < 0.001.

**FIGURE 2:**
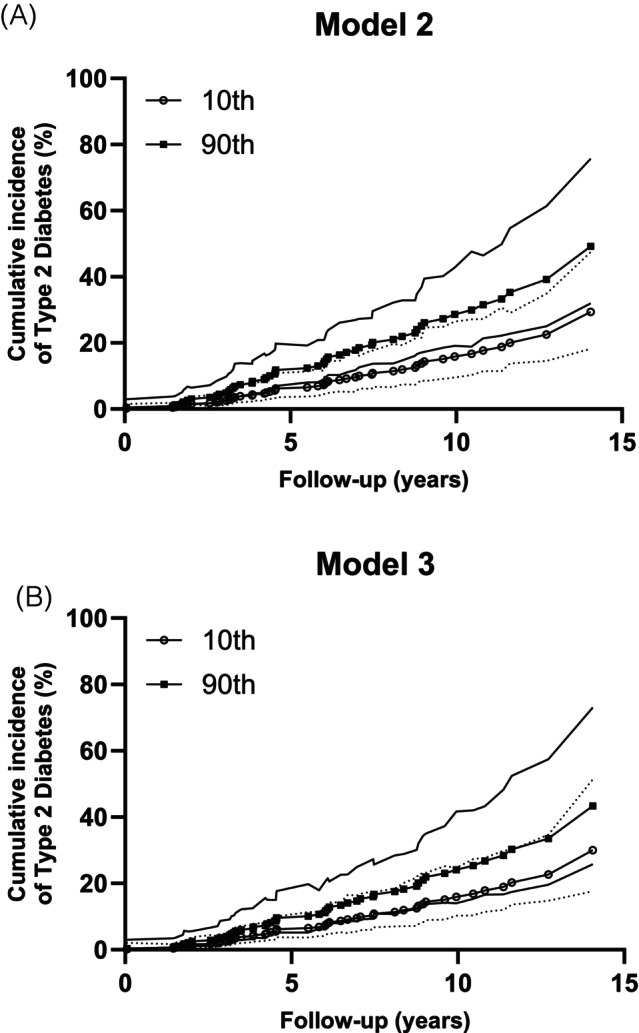
Predicted cumulative incidence of type 2 diabetes with 95% confidence intervals at 10th (open circles) and 90th (closed squares) percentiles of corrected anion gap when (A) accounting for age, sex, body fat%, and plasma 2‐h glucose, and (B) accounting for age, sex, body fat (%), and plasma 2‐h glucose and M‐low (log_10_).

Table 3 presents Cox proportional hazards models of CAG and incident type 2 diabetes. CAG was associated with type 2 diabetes onset in the unadjusted model (model 0; HR: 1.38, 95% CI: 1.07–1.80, *p* = 0.02). The results persisted when adjusted for age, sex, body fat percentage, and 2‐h plasma glucose (model 2; HR: 1.32, 95% CI: 1.01–1.74, *p* = 0.044). However, CAG was no longer statistically significant after adjusting for M‐low (model 3; HR: 1.22, 95%CI: 0.92–1.61, *p* = 0.17). As visualised in Figure 2, higher CAG was associated with higher predicted cumulative incidence of type 2 diabetes in model 2 (Figure 2A) but not after further adjusting for M‐low (Figure 2B).

**TABLE 3:** Cox proportional hazards models examining association between corrected anion gap and type 2 diabetes.

Model adjustments	HR	(95% CI)	*p*
Unadjusted model 0			
Corrected anion gap	**1.38**	(1.07–1.80)	0.02
Adjusted model 1			
Corrected anion gap	**1.37**	(1.04–1.80)	0.02
Age	**1.13**	(1.00–1.75)	0.0496
Sex	0.91	(0.43–1.95)	0.81
Body fat %	**1.61**	(1.08–2.4)	0.02
Adjusted model 2			
Corrected anion gap	**1.32**	(1.01–1.74)	0.044
Age	1.15	(0.86–1.54)	0.36
Sex	0.83	(0.39–1.79)	0.64
Body fat %	1.47	(0.97–2.22)	0.07
Plasma glucose, 2‐h	**1.78**	(1.30–2.44)	0.0003
Adjusted model 3			
Corrected anion gap	1.22	(0.92–1.61)	0.17
Age	1.12	(0.83–1.50)	0.47
Sex	1.25	(0.52–2.98)	0.62
Body fat %	1.16	(0.72–1.89)	0.55
Plasma glucose, 2‐h	**1.61**	(1.15–2.24)	0.005
M‐low	0.58	(0.32–1.03)	0.06

*Note*: All continuous variables in models were standardised to mean = 0, SD = 1. Bolded coefficients were statistically significant (*p* < 0.05). For sex, male is reference group.

Bicarbonate and AG were not associated with incident type 2 diabetes in unadjusted and adjusted Cox proportional hazards models (Tables S1 and S2).

Because circulating albumin constitutes a large proportion of unmeasured anions, an increase or decrease in albumin can alter the anion gap.^28^ Additionally, past research has found an association between increased albumin and higher M, when accounting for age and sex.^17^ Given these past results, we performed an additional analysis examining the impact of albumin on M‐low and type 2 diabetes risk. The association between albumin and M‐low (log) was significant (*r* = 0.16, *p* = 0.0051) and remained significant in the partial *r* accounting for age and sex (adjusted *r* = 0.15, *p* = 0.01), with the association becoming null following the inclusion of % body fat (adjusted *r* = 0.05, *p* = 0.44). Cox proportional hazard models found an association between increased albumin and lower likelihood of type 2 diabetes in unadjusted models (HR: 0.57, 95% CI: 0.43–0.83, *p* = 0.0003), and adjusting for CAG (HR: 0.61, 95% CI: 0.44–0.77, *p* = 0.0017). On the other hand, CAG was not significantly associated with type 2 diabetes risk in the model adjusting for albumin (HR: 1.22, 95% CI: 0.93–1.61, *p* = 0.16, see Table S3).

## 4 DISCUSSION

Complementary analyses in the current study delineated the relationships between acid accumulation, insulin resistance and type 2 diabetes. In healthy adults without diabetes or kidney disease, markers of acid accumulation (i.e., lower bicarbonate, higher AG, and higher CAG) were cross‐sectionally associated with lower insulin sensitivity independent of important confounders including age, sex, adiposity and eGFR. In prospective analysis, higher CAG was associated with incident type 2 diabetes in Cox proportional hazards models adjusted for age, sex, body fat % and 2‐h plasma glucose concentrations. This association was no longer significant in models adjusted for insulin sensitivity, indicating that the association with onset of future diabetes may be confounded by measures of insulin resistance.

Strengths of the current study are the measurement of insulin sensitivity using a gold standard method (i.e., glucose‐clamp technique) in a large sample of participants with acid accumulation markers and longitudinal follow up visits using an OGTT. Markers of acid accumulation were correlated with lower insulin sensitivity at both maximal and submaximal insulin stimulation (M‐high and M‐low, respectively). As insulin has concentration‐dependent saturable actions to increase whole‐body glucose uptake, the two infusion rates decrease the likelihood of missing potential differences among insulin‐resistant individuals.^29^ These findings represent an advance in this area as prior literature used surrogate measures of insulin sensitivity instead of reference methods^13^ or were small experimental studies manipulating acid–base balance.^12^ Previous prospective studies demonstrating the link between acid markers and diabetes risk have not accounted for insulin resistance (using any method), have not used a gold standard method to measure insulin resistance, or based diabetes onset on self‐report only.^14^, ^15^, ^16^

The mechanism linking acid accumulation and insulin resistance may be due to increased pH in the interstitial fluid which is much less buffered (compared with blood) and where most interactions between insulin and its receptor occur.^5^ Decreased pH in interstitial fluid may lead to inhibition of phosphofructokinase and dysregulated insulin signalling at the receptor level and downstream pathways (e.g., Akt phosphorylation) as indicated by prior experiments in rodent adipocytes and muscle.^7^, ^8^, ^9^, ^10^ In addition, lactate may be a source of fixed acid contributing to acidosis. Lactate accumulation in obesity, possibly via lactate overproduction by enlarged adipocytes, may lead to inflammation by release of proinflammatory cytokines, leading to systemic insulin resistance.^30^ Future studies may consider measuring lactate and interstitial pH.

The reasons that AG and bicarbonate were not while CAG was associated with incident diabetes is uncertain. As circulating albumin constitutes a large portion of unmeasured anions, its decrease or increase can alter anion gap. CAG adjusts for albumin concentrations and may be more effective at identifying acidosis (e.g., hyperlactaemia) compared with uncorrected AG, leading some researchers to recommend routine calculation of CAG as a part of standard clinical laboratory reporting.^28^ That CAG is no longer an independent predictor of diabetes after albumin is included in the model indicates that albumin is the important factor driving the relationship with diabetes. Furthermore, albumin gene expression is positively controlled by insulin.^31^ In the current study population with high insulin resistance, decreased albumin is associated with increased risk of diabetes as previously reported.^17^ Thus, CAG may be a better reflection of insulin resistance via diminished albumin gene expression.

The reasons for inter‐individual differences in accumulating acid are unclear. Diet may be a factor influencing these markers of acid accumulation. The composition of the contemporary Western diet induces high net endogenous acid production due to its effects on the balance of acid and alkali precursors consumed.^32^ Proteins, especially from animal sources which have increased content of sulphur‐containing amino acids, are the most important contributors to metabolic production of nonvolatile acid (i.e., that cannot be exhaled as carbon dioxide), while dietary potassium salts such as those in fruits and vegetables decrease net endogenous acid production.^33^ The impact of net endogenous acid production on chronic kidney disease is increasingly recognised such that reduction of dietary acid load is now recommended in the clinical practice guidelines of the National Kidney Foundation's Kidney Disease Quality Initiative to prevent declines in residual kidney function even in those without diabetes.^34^ Thus, targeting acid accumulation to reduce insulin resistance, such as through dietary intervention on dietary acid load, may decrease the risk of both diabetes and kidney disease.

Several limitations should be acknowledged. First, interstitial fluid pH was not measured. Although interstitial pH was decreased in a murine model of diabetes,^6^ it is unknown if those with increased acid accumulation markers in the current human study also have decreased interstitial fluid pH. Second, the study involved Indigenous Americans living in the southwestern United States, a population at high risk for the development of diabetes, and results may not be generalisable to other populations. However, the underlying pathophysiology of diabetes (i.e., insulin resistance) is shared between this and other populations alike. Third, most participants had acidosis values within the clinical normal range, and more severe acidotic states were not examined. Thus, the current study suggests that variation within the normal range may influence insulin resistance. In support of this possibility, mild acidosis, induced experimentally, has been shown to impact insulin resistance.^12^ Our biomarkers capture chronic, low‐grade acid accumulation. During exercise, however, transient rises in lactate and accompanying protons activate AMPK and increase GLUT4 translocation, thereby enhancing skeletal muscle glucose uptake.^35^, ^36^ This acute, beneficial effect contrasts with the chronic hyperlactaemia linked to systemic insulin resistance. Future work should consider both timescales when interpreting acid‐base markers. Finally, we must acknowledge that our markers of acidosis [decreased plasma bicarbonate, increased anion gap (AG) and corrected anion gap (CAG)] do not identify or distinguish between various acid sources (e.g., lactate vs. ketoacids). Future research should consider including markers that capture physiological and pathological diversity in acid production.

In summary, markers of higher acid accumulation (lower bicarbonate, higher AG and higher CAG) were associated with lower insulin sensitivity in adults without diabetes. CAG was associated with an increased risk of incident type 2 diabetes. These findings support interventions targeting acid accumulation to prevent insulin resistance and its complications, including type 2 diabetes.

## FUNDING INFORMATION

Support for the research was provided by the Intramural Research Program of the U.S. National Institutes of Health, National Institute of Diabetes and Digestive and Kidney Diseases.

## CONFLICT OF INTEREST STATEMENT

The authors declare that they have no conflicts of interest.

## PEER REVIEW

The peer review history for this article is available at https://www.webofscience.com/api/gateway/wos/peer-review/10.1111/dom.70037.

## Supporting information


**Table S1.** Cox proportion hazards models examining association between bicarbonate and type 2 diabetes.

**Table S2.** Cox proportional hazards models examining association between anion gap and type 2 diabetes.

**Table S3.** Cox proportional hazard models examining the association between albumin and type 2 diabetes.

## Data Availability

The datasets used and/or analysed during the current study are available from the corresponding author on reasonable request and approval.
