# Supplementary material for: Acidosis is associated with lower insulin sensitivity and incident type 2 diabetes in indigenous Americans: A prospective cohort study
Source: Diabetes Obes Metab. 2025 Aug 18;27(11):6440–8. doi: 10.1111/dom.70037 (PMC12515791; doi:10.1111/dom.70037)
Supplement: Supplementary file 1 — Table S1. Cox proportion hazards models examining association between bicarbonate and type 2 diabetes. [file DOM-27-6440-s003.docx]

**Supplemental Table 1.** Cox proportion hazards models examining association between bicarbonate and type 2 diabetes

| **Model adjustments** | **HR** | **(95% CI)** | ***p*** |
| --- | --- | --- | --- |
| **Unadjusted model 0** |  |  |  |
| Bicarbonate | 0.88 | (0.67 – 1.16) | 0.37 |
| **Adjusted model 1** |  |  |  |
| Bicarbonate | 0.99 | (0.73 – 1.34) | 0.93 |
| Age | **1.37** | (1.03 – 1.81) | 0.03 |
| Sex | 0.91 | (0.42 – 1.94) | 0.80 |
| Body fat % | **1.59** | (1.07 – 2.38) | 0.02 |
| **Adjusted model 2** |  |  |  |
| Bicarbonate | 1.00 | (0.74 – 1.35) | 0.99 |
| Age | 1.19 | (0.89 – 1.60) | 0.23 |
| Sex | 0.81 | (0.38 – 1.75) | 0.59 |
| Body fat % | 1.45 | (0.96 – 2.20) | 0.08 |
| Plasma glucose, 2-h | 1.81 | (1.33 – 2.48) | 0.0002 |
| **Adjusted model 3** |  |  |  |
| Bicarbonate | 1.01 | (0.75 – 1.37) | 0.96 |
| Age | 1.15 | (0.85 – 1.54) | 0.37 |
| Sex | 1.32 | (0.55 – 3.17) | 0.53 |
| Body fat % | 1.10 | (0.68 – 1.78) | 0.71 |
| Plasma glucose, 2-h | **1.61** | (1.16 – 2.24) | 0.0046 |
| M-low | **0.52** | (0.29 – 0.91) | 0.0227 |
| All continuous variables in models were standardized to mean = 0, SD = 1. Bolded coefficients were statistically significant (p < 0.05). For sex, male is reference group. | | | |
