# Supplementary material for: Acidosis is associated with lower insulin sensitivity and incident type 2 diabetes in indigenous Americans: A prospective cohort study
Source: Diabetes Obes Metab. 2025 Aug 18;27(11):6440–8. doi: 10.1111/dom.70037 (PMC12515791; doi:10.1111/dom.70037)
Supplement: Supplementary file 2 — Table S2. Cox proportional hazards models examining association between anion gap and type 2 diabetes. [file DOM-27-6440-s001.docx]

**Supplemental Table 2.** Cox proportion hazards models examining association between anion gap and type 2 diabetes

| **Model adjustments** | **HR** | **(95% CI)** | ***p*** |
| --- | --- | --- | --- |
| **Unadjusted model 0** |  |  |  |
| Anion Gap | 1.21 | (0.92 – 1.59) | 0.17 |
| **Adjusted model 1** |  |  |  |
| Anion Gap | 1.25 | (0.94 – 1.66) | 0.12 |
| Age | **1.34** | (1.01 – 1.78) | 0.04 |
| Sex | 0.91 | (0.43 – 1.93) | 0.80 |
| Body fat % | **1.64** | (1.10 – 2.45) | 0.01 |
| **Adjusted model 2** |  |  |  |
| Anion Gap | 1.23 | (0.93 – 1.64) | 0.15 |
| Age | 1.16 | (0.87 – 1.56) | 0.31 |
| Sex | 0.82 | (0.39 – 1.77) | 0.62 |
| Body fat % | 1.50 | (0.99 – 2.26) | 0.06 |
| Plasma glucose 2-h | **1.81** | (1.32 – 2.48) | 0.0002 |
| **Adjusted model 3** |  |  |  |
| Anion Gap | 1.13 | (0.84 – 1.50) | 0.42 |
| Age | 1.13 | (0.84 – 1.52) | 0.42 |
| Sex | 1.28 | (0.54 – 3.06) | 0.57 |
| Body fat % | 1.15 | (0.70 – 1.87) | 0.59 |
| Plasma glucose 2-h | **1.61** | (1.16 – 2.25) | 0.005 |
| M (logmlow) | **0.54** | (0.31 – 0.97) | 0.04 |
| All continuous variables in models were standardized to mean = 0, SD = 1. Bolded coefficients were statistically significant (p < 0.05). For sex, male is reference group. | | | |
