# Supplementary material for: Acidosis is associated with lower insulin sensitivity and incident type 2 diabetes in indigenous Americans: A prospective cohort study
Source: Diabetes Obes Metab. 2025 Aug 18;27(11):6440–8. doi: 10.1111/dom.70037 (PMC12515791; doi:10.1111/dom.70037)
Supplement: Supplementary file 3 — Table S3. Cox proportional hazard models examining the association between albumin and type 2 diabetes. [file DOM-27-6440-s002.docx]

**Supplemental Table S3.** Cox proportional hazard models examining the association between albumin and type 2 diabetes.

| **Model adjustments** | **HR** | **(95% CI)** | | ***p*** |
| --- | --- | --- | --- | --- |
| **Unadjusted Model 0** |  |  |  |  |
| Albumin | **0.57** | (0.43 - 0.77) | | 0.0003 |
| **Unadjusted Model 1** |  |  |  |  |
| Albumin | **0.61** | (0.44 - 0.83) | | 0.0017 |
| Corrected anion gap | 1.22 | (0.93 - 1.61) | | 0.16 |
| **Unadjusted Model 2** |  |  |  |  |
| Albumin | 0.70 | (0.49 - 1.00) | | 0.052 |
| Corrected anion gap | 1.09 | (0.81- 1.47) | | 0.57 |
| Age | 1.10 | (0.81 - 1.49) | | 0.54 |
| Sex | 1.18 | (0.49 - 2.84) | | 0.71 |
| Body fat % | 1.06 | (0.64 - 1.75) | | 0.83 |
| Plasma glucose, 2-h | **1.55** | (1.12 - 2.16) | | 0.0084 |
| M-low | 0.58 | (0.33 - 1.03) | | 0.06 |
| All continuous variables in models were standardized to mean = 0, SD = 1. Bolded coefficients were statistically significant (p < 0.05). For sex, male is reference group. | | | | |
